# Divergent age-related changes in parasite infection occur independently of behaviour and demography in a wild ungulate

**DOI:** 10.1098/rstb.2023.0508

**Published:** 2024-10-28

**Authors:** Gregory F. Albery, Adam Z. Hasik, Sean Morris, Alison Morris, Fiona Kenyon, David McBean, Josephine M. Pemberton, Daniel H. Nussey, Josh A. Firth

**Affiliations:** ^1^ Institute of Ecology and Evolution, University of Edinburgh, Edinburgh EH9 3FL, UK; ^2^ Department of Biology, Georgetown University, Washington, DC 20057, USA; ^3^ Leibniz Institute of Freshwater Ecology and Inland Fisheries, Berlin 12587, Germany; ^4^ School of Natural Sciences, Trinity College Dublin, Dublin, D02 PN40, Ireland; ^5^ Moredun Research Institute, Penicuik EH26 0PZ, UK; ^6^ Department of Biology, University of Oxford, Oxford OX1 3SZ, UK; ^7^ School of Biology, University of Leeds, Leeds LS2 9JT, UK

**Keywords:** ageing, infectious disease, parasites, behaviour, disease ecology, wild animal

## Abstract

As animals age, they exhibit a suite of phenotypic changes, often including reductions in movement and social behaviour (‘behavioural ageing’). By altering an individual’s exposure to parasites, behavioural ageing may influence infection status trajectories over the lifespan. However, these processes could be confounded by age-related changes in other phenotypic traits, or by selective disappearance of certain individuals owing to parasite-induced mortality. Here, we uncover contrasting age-related patterns of infection across three helminth parasites in wild adult female red deer (*Cervus elaphus*). Counts of strongyle nematodes (order: Strongylida) increased with age, while counts of liver fluke (*Fasciola hepatica*) and tissue worm (*Elaphostrongylus cervi*) decreased, and lungworm (*Dictyocaulus*) counts did not change. These relationships could not be explained by socio-spatial behaviours, spatial structuring, or selective disappearance, suggesting behavioural ageing is unlikely to be responsible for driving age trends. Instead, social connectedness and strongyle infection were positively correlated, such that direct age–infection trends were directly contrasted with the effects implied by previously documented behavioural ageing. This suggests that behavioural ageing may reduce parasite exposure, potentially countering other age-related changes. These findings demonstrate that different parasites can show contrasting age trajectories depending on diverse intrinsic and extrinsic factors, and that behaviour’s role in these processes is likely to be complex and multidirectional.

This article is part of the discussion meeting issue ‘Understanding age and society using natural populations’.

## 1. Introduction

An individual’s disease status depends on a combination of its exposure and susceptibility to parasites [1,2]. Exposure is broadly a function of an individual’s social and spatial behaviour within the context of a population, and between- and within-individual variation in behaviour can have important consequences for infectious disease status [3–5]. In humans and wild animals, individuals alter their behaviour as they age [6,7], with a series of general changes characterized by reduced movement [8–10] and sociality [10,11], and specifically a tendency towards positive interactions with specific individuals known as ‘social selectivity’ [11–14]. Because these processes influence the way that individuals contact each other, they could affect rates of exposure to pathogens, and therefore their infection probability [6]. Nevertheless, since behaviour has yet to be linked to age-related changes in infection in a given population, the role of behavioural ageing in driving infection dynamics remains unclear [6].

Behaviour could drive age-related changes in infection status through a series of mechanisms (see [6] for a review). For example, individuals could alter their feeding locations as they age, which could move them into areas that are more or less likely to support environmental parasites [5], or it could result in lower-quality resource intake, driving weaker immunity and therefore greater susceptibility to infection [15]. Similarly, ageing individuals could become more socially isolated, potentially driving decreased exposure to directly transmitted parasites [7,10]. More subtly, if ageing individuals tend to prefer a few close associates over socializing broadly (i.e. showing increasing selectivity), this could drive an increase in modularity, with complex outcomes for epidemiological dynamics [6,16]. However, age also alters other phenotypic and demographic changes that could complicate these relationships. For example, ageing individuals experience a suite of physiological changes [17], many of which affect the immune system (i.e. ‘immunosenescence’ [18–20]). Because these changes often result in increased susceptibility to infection, the conventional wisdom is that individuals will exhibit a greater prevalence or burden of parasites as they senesce [20–22]. Alternatively, individuals may acquire adaptive immunity to certain parasites as they become exposed, potentially leading to an increase in immunity to these particular parasites [23]. Additionally, because parasites often exert survival costs on their hosts, more heavily infected individuals may be more likely to die—a process known as ‘selective disappearance’—which could produce a negative age–infection trend at the population level and may bias estimates of within-individual ageing patterns [24,25]. The emergent pattern of infection status over the lifespan will depend on a combination of these factors.

Given these combined behavioural, immunological, and demographic changes, ageing individuals’ infection statuses could be asynchronous and divergent for different parasite taxa, leading to an age-related shift in parasite community composition. This possibility is supported by the literature on observed age–infection relationships, which comprises a wide diversity of positive, negative, and nonlinear changes in prevalence and intensity of infection (e.g. [26–31]); however, most such studies focus on one parasite taxon, and it is therefore unclear how often parasites show divergent age-related trends within a population. These studies are likewise often cross-sectional rather than longitudinal (i.e. they do not follow the same known individuals through time), and are therefore unable to identify and extricate selective disappearance effects ([24,25]; but see [26,27,30]). This is an especially important gap in our understanding, particularly given that parasites are generally defined by their ability to cause harm to their hosts [6,32] and will therefore likely drive patterns of disappearance. Additionally, because studies rarely model variable age–infection relationships within a given population (which requires longitudinal data), it is unclear how these processes drive variable infection trajectories over the lifespan [6]. Contrasting age trajectories for different parasites in the same individuals within the same population may help to untangle the mechanisms underlying age–infection trends more broadly.

Here, we examine how different helminth parasite counts change over the lifespan in a long-term study population of wild red deer (*Cervus elaphus*), in which female deer are monitored from birth until their death, generally at least a decade later. Building on a rigorous behavioural censusing operation [33] in a society with well understood spatial structuring [34], studies have shown that female deer strongly alter their foraging and social behaviour as they age [9,10]. Specifically, they reduce their home range sizes [9] as well as moving towards areas of lower density at the periphery of the population, and become less socially connected [10]. The deer also feature high-resolution, individually tied egg and larval counts of multiple helminth parasite taxa. These parasites infect individuals throughout their lives without inducing full immunity, and therefore exist at high prevalence in the population, but with substantial within- and between-individual variation that enables testing of a wide range of ecological questions [35]. Counts of these parasites fluctuate seasonally [35] and are influenced by allocation of resources to reproduction [36], as well as having strong costs in terms of survival and reproduction [37]. Combining these sources of information, this population is well suited to examining long-term age trajectories of infection by multiple parasites, and the possible role of behaviour and demography in driving them. Specifically, we ask (i) how counts of multiple helminth parasites change over the lifespan; (ii) whether these counts are influenced by spatial and social behaviours governing rates of exposure; and (iii) whether these behaviours could explain or counteract the age-related changes we see.

## 2. Methods

### (a) Study population

The study population was the individually monitored Isle of Rum red deer. This unmanaged wild population has been studied since 1973 [33], with regular faecal parasite sampling since 2016 [35]. The deer are censused 40 times a year, with individuals known by name and individually marked using a combination of coloured and patterned collars, tags, and ear punches. When identified in a census, an individual’s location (to the nearest hectare) is recorded, providing it with an easting and northing location in two-dimensional space; further, groups of deer are identified in the course of censusing and taken by the field worker to be associating, forming the basis for the social network pipeline described below. The type of vegetation each deer is on is noted.

The deer give birth in May and June, and daily censuses over the calving period allow >90% of calves to be caught, tagged and weighed. The deer year runs from 1 May, and individuals are assigned an age in years based on the deer year they were born in; for example, all individuals turn 1 year old on 1 May the year after they were born. Forty study area censuses per year allow us to keep track of each individual’s life history, and individuals have known death dates, generally to within one month, and often to the day, allowing accurate quantification of mortality. Following our previous related work in this system [10,34], here we assess mature females (3 years and older), as these are the best-understood age and sex class, with the largest available dataset; young males disperse and few adult males live in the study area, and so males are less well sampled. Female reproductive status in any year was coded as either ‘none’ (did not give birth that year), ‘summer’ (gave birth, but the calf died before 1 October), or ‘winter’ (gave birth, but the calf died during its first winter, or was reared through its first winter). This categorization is based on the relative costs of reproduction, which are observed to be high in individuals whose calf survives to the winter, regardless of whether the calf then survives to the spring; these costs are reflected in terms of both parasitism and fitness [36–38].

### (b) Parasitology

We have previously described our parasitology monitoring regime in detail [35]. Briefly, three times a year (late April, August and November), for two weeks at a time, we observe the deer intensively to collect faecal samples from as many individuals as possible. After observing an individual defaecating, we collect the sample as soon as possible into a resealable plastic bag, and at the end of the day, we homogenize it, and store it anaerobically (i.e. with the bag sealed) in a fridge at approximately 4°C until counting. By observing the individual and noting the location of the defaecation event itself, coupled with collection within a short period (generally within an hour and most often within 10–20 min), we are able to tie samples to known individuals.

We counted gastrointestinal helminth parasite propagules in these samples using a variety of techniques. We counted strongyle nematode (order: Strongylida) eggs within three weeks of collection using a salt flotation–centrifugation technique, where a gram of homogenized faecal matter was mixed with saturated salt solution and the mixture homogenized, causing the eggs of a selection of parasites to rise to the surface, where they could be easily counted [35]. Liver fluke (*Fasciola hepatica*) eggs were counted using a sedimentation technique, where a weighed amount of faecal matter was mixed with a large amount of sediment and allowed to settle over 3 min, and the supernatant removed via vacuum suction. Finally, tissue worm (*Elaphostrongylus cervi*) and lungworm (*Dictyocaulus* sp.) larvae were counted using a Baermannization technique. In this method, a weighed amount of faecal matter was wrapped in porous cloth and submerged in water for 24 h to allow the mobile larvae to escape, which were then reduced in volume by vacuum suctioning and preserved for counting. All techniques were accurate to at least 1 egg or larva per gram. These different assays were required because of the different physical properties of the propagules: strongyle eggs float in saturated salt solution, whereas fluke eggs are too heavy and must be sedimented, while tissue worm and lungworms are alive and possible to isolate using their movement behaviour. Our salt flotation also detected a number of other parasites (described in [35]), but they were present at low prevalence (<10%) in adult females, and therefore we were less able to analyse how they changed with age.

Samples were collected between August 2016 and April 2021. Where multiple samples were collected for a given individual in a given sampling trip, we took the mean of the counts to leave a maximum of one count per individual per sampling trip. We did so because there were relatively few within-season repeats, and they were restricted to the beginning of the study (*n* = 654 repeats). Our final dataset included *N*_s_ = 1449 measurements taken from *N*_i_ = 210 individuals; some assays were not completed for all samples, leaving *N*_s_ = 1433 *F*. *hepatica* measurements and *N*_s_ = 1126 *E*. *cervi* and *Dictyocaulus* measurements taken from *N*_i_ = 209 individuals. The numbers of samples per individual and per sampling trip are displayed in electronic supplementary material, table S2.

### (c) Behavioural metrics

We examined how an individual’s behaviour was associated with its parasite burden. Building from our prior findings that individuals alter a suite of socio-spatial behaviours as they age, we selected a series of behaviours to test. All such behaviours are expected to influence some element of exposure to parasites, involving either movement to different areas on the landscape or interactions with other individuals. We used all census observations of each individual in each year, including adults and juveniles. We chose to include juveniles in the social network as they are heavily infected with parasites [35] and could therefore play an important role in infecting older individuals. The behavioural metrics include:

*Social network metrics*: We constructed social networks as previously described [10,34]. Social connections were judged by field workers based on a spatially parameterized ‘gambit of the group’ approach, where individuals within a certain distance of each other were taken to be socializing (refer [10,34] for details), as described above. First, we took the average group size for each individual across the year. Next, for each year, we constructed networks based on these associations, which we corrected for observation bias using the simple ratio index [39] such that each dyad’s connection was scaled between 0 (never seen together) and 1 (never seen apart). We then calculated two network metrics: degree centrality (i.e. the number of individuals an individual was seen with over the course of a year) and strength centrality (i.e. the summed weighted connections to all individuals over the year).

*Local population density*: We calculated local density using a previously described pipeline for this population [10,34], using all observations of each individual in each year, including both adults and juvenile individuals. This approach uses a kernel density estimator, taking individuals’ annual centroids and fitting a two-dimensional smooth to the distribution of the data, producing a two-dimensional spatial distribution of the population. Individuals are then assigned a local density value based on their location on this kernel.

*Spatial behaviour metrics*: we included several metrics that quantitatively described an individual’s spatial behaviour in the study area, all of which have been shown to change with age [10]. These included: population centroid distance (the distance from the overall mean location of the population, which increases with age); graze type (the proportion of sightings in which an individual was seen on high-quality grazing, which decreases with age); and home range area (built based on each individual’s density distribution, which decreases with age).

*Time lag*: We examined how annual behaviour metrics from deer year *t* influenced parasite infection in deer year *t* + 1. To put this in terms of calendar years, we examined how an individual’s behaviour from 1 May in year *t* to 30 April in year *t* + 1 affected its parasite burden in August in year *t* + 1, November in year *t* + 1 and April in year *t* + 2.

Although a relatively coarse annual measure of behaviour, individual-level repeatability of annual social network positions is high [34], as is repeatability of annual measures of spatial fidelity and home range size [9,40], and previous work has shown these measures to be ecologically relevant for individuals [10,34]. Using the previous deer year’s social network also allowed us to accommodate the time lag of the influence of social connections on parasite burden (e.g. including parasites’ time to development and maturation and egg production, which generally take months to stabilize) and allowed us to avoid confounding produced by analysing an individual’s social connectedness in a given deer year with its concurrent and earlier parasite infection status, and possible reverse causality emerging from, e.g., avoidance responses [41]. That is, including behavioural measures taken in deer year *t* in models examining parasite infection through deer year *t* would involve including behavioural observations from post parasite sampling; because behaviours often change in response to infection, and often with protective consequences that decrease the risk of infection [42]; this could drive complex and counterintuitive relationships with parasitism that we were not intending to test. Finally, behaviour of the deer in this population is highly seasonal [33], as is parasite infection [35]; using sub-annual measures of infection that differed between seasons might risk strong confounding between behaviour and infection. As such, we judge our annual measures to be a reliable and parsimonious indicator of social and spatial behaviour with relevance to the risk of parasite transmission over the lifespan.

### (d) Models

Our dataset included 1449 measures of parasite counts in 210 individual deer, spread across 5 deer years and 15 collecting seasons. To identify age-related changes in parasite burden and determine how they might arise, we fitted a selection of generalized linear mixed models (GLMMs) using the integrated nested Laplace approximation (INLA) in R [43]. INLA is a deterministic Bayesian algorithm that allows fitting of spatially distributed random effects (stochastic partial differentiation equation (SPDE) effects, see below) to account for spatial autocorrelation in the response variable [44]. All models were fitted with uninformative default priors. Models were checked by simulating from the model posteriors, and inspecting the predicted against the observed values and examining them for uneven patterns. We calculated *p*-values from the posteriors using the ‘inla.pmarginal’ function, providing the probability of generating a result that overlapped with zero from the distribution. For all models, continuous predictors were scaled to have a mean of 0 and a standard deviation of 1 before analysis. The model sets we used were as follows.

*Base models*: First, we fitted models to understand individual age trajectories of parasitism in the population. We examined each parasite count as a response variable with a negative binomial specification, given their strongly overdispersed distribution. We fitted explanatory variables including year (factor with five levels: deer years? 2016–2020); season (factor with three levels: summer, autumn and spring); reproductive status (factor with three levels: none, summer and winter); age (continuous covariate, range 3–24, mean 7.9). We ran these models both without and with a random effect of individual identity, to examine how controlling for among-individual variation impacted our estimates of age effects. Using individual identity in this way can help to distinguish within-individual ageing processes versus between-individual demographic processes [24]; fitting an ID effect and seeing the disappearance of an age effect would imply that age was only associated with infection at the between-individual level.

*Social models*: Second, to identify the effects of a given behaviour on infection—and the effects of incorporating the said effect on age–infection relationships—we ran a series of models, each of which added a behavioural metric to the base model. We then investigated the mean estimate and 95% credible interval of this behavioural metric effect, and examined the impact that its inclusion had on the age effect estimate to ask whether behaviour could be responsible. We fitted behavioural metrics in a piecewise fashion—rather than adding them all at the same time—because the age-related changes tend to manifest as correlated socio-spatial behaviour syndromes [10]. We excluded counts from the autumn, because their low values precluded fitting as explanatory variables in our models. Adding all at the same time would risk substantial collinearity, and fitting them one at a time allowed us to test our hypotheses effectively.

*Spatial models*: For each model, to identify whether our results were affected by spatial autocorrelation, we added a spatially distributed SPDE effect [44–46] in INLA. This effect uses each individual’s average annual easting and northing to model how spatial proximity drove individuals to have similar parasite counts, according to Matern covariance. Fitting this effect had three purposes: (i) by comparing the fit of the spatial model with the base model, we could identify whether the parasite counts were significantly spatially autocorrelated; (ii) by comparing the model estimates we could identify whether this spatial autocorrelation was affecting our conclusions; and (iii) by plotting the effect in space we could identify spatial hotspot and coldspot of infection [46]. To assess model fit, we used deviance information criterion (DIC), with a cutoff of −2ΔDIC to distinguish between competitive models.

*Survival models*: Often, ageing models incorporate fixed effects of longevity to examine selective disappearance of certain individuals [24]. We were unable to do this with our dataset, as it spanned 5 years running to the present; because many individuals were yet to die, we did not have known longevity values for many of the data points, which reduced our models’ power in this context. As such, to provide an approximate answer to this question, we fitted binomial survival models following the previous methodology [37] to examine whether parasites were likely to be causing annual mortality in adult females (i.e. the same dataset we were testing for age–infection associations), and therefore might be producing observed age–infection relationships. With observations from each individual : deer year combination as the unit of investigation, we fitted overwinter survival (0/1) as a response variable, with explanatory variables including: deer year; reproductive status; age; and a random effect of individual identity, all as described above. We sequentially added each parasite count (log(*X* + 1)-transformed) as an explanatory variable, one at a time, to investigate whether they correlated with subsequent survival. In our dataset, there was an 89.1% annual survival rate across the 6 years of sampling; of our 208 individuals in the survival models, 83 (40%) died. We note that this is a relatively crude way of assessing selective disappearance effects that was necessitated by our dataset; depending on the effects shown by the mortality assessments, we may or may not be able to infer an effect of selective disappearance using such an analysis. However, this approach to detecting survival effects has high statistical power and has been used previously to detect strong survival effects of parasitism [37], which is the central hypothesized cause of selective disappearance in this context; this article expands on this analysis by including more data, confirming the patterns using an expanded dataset and differently parameterized models, and by testing multiple pathogens.

## 3. Results

**Figure 1.**
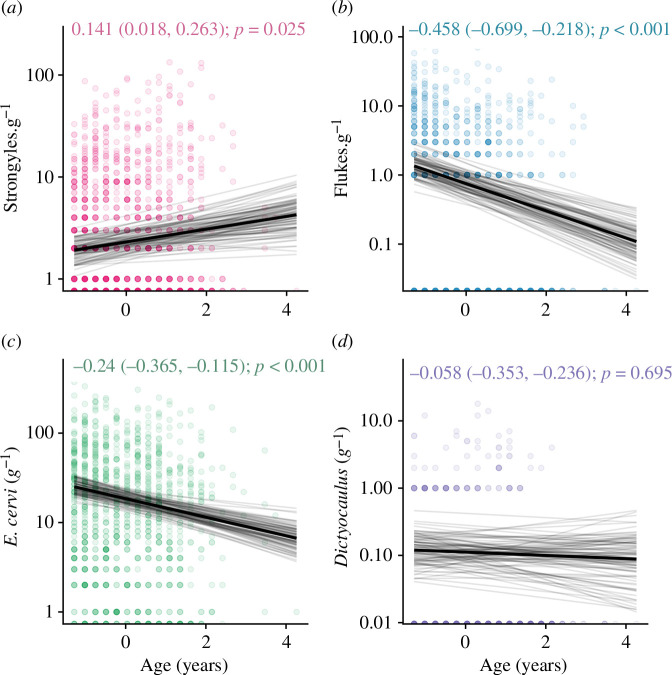
Age-related changes in infection with four helminth parasites in wild red deer. The *x*-axis represents age in years. Number of (*a*) strongyle eggs per gram; (*b*) fluke eggs per gram; (*c*), *Elaphostrongylus cervi* larvae per gram; (*d*) *Dictyocualus* larvae per gram. Taken from the best-fitting models, the black line represents the mean of the posterior distribution for the age effect estimate; the light grey lines are 100 random draws from the posterior to represent uncertainty. The age effect estimate, credibility intervals and *p*-values are given at the top of each panel. The points represent individual samples, with transparency to allow visualization of overplotting. The *y*-axis has been log_10_-transformed; zero-counts (which are not possible to display on this log scale) are displayed at the bottom of the graph.

**Figure 2.**
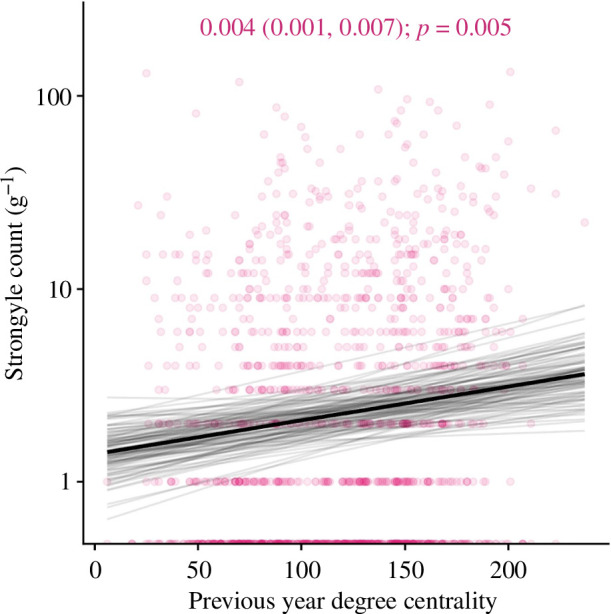
Association between social connectedness (degree centrality) in the previous year and strongyle nematode count in wild red deer. The *x*-axis shows number of contacts; the *y*-axis shows number of eggs per gram. Taken from the spatial model, the black line represents the mean of the posterior distribution for the age effect estimate; the light grey lines are 100 random draws from the posterior to represent uncertainty. The degree effect estimate, credibility intervals and *p*-values are given at the top of the figure. The points represent individual samples, with transparency to allow visualization of overplotting. The figure has been cropped to the distribution of the fitted lines to help visualizing the model fits, so some points outside this range have been excluded from the figure.

We found substantial contrasting age–infection relationships for three out of four parasites: there were small positive associations between age and strongyle count (figure 1*a*; mean effect estimate: 0.138, lower 95% credibility estimate: 0.014, upper 95% credibility estimate: 0.261, *p* = 0.029), and moderate negative associations between age and liver fluke *F. hepatica* (figure 1*b*; 0.372, −0.605, −0.141, *p* = 0.002) and tissue worm (*E. cervi*) count (figure 1*c*; −0.251, −0.364, −0.14, *p* < 0.001). *Dictyocaulus* lungworms, meanwhile, showed no relationship with age (figure 1*d*; *p* > 0.05). All effect estimates and 95% credibility intervals are derived from the mean of the posterior effect distribution; we report estimates here and in the model effects plots in units of standard deviations, but to aid interpretation in the scale of the lifespan, in figures 1 and 2 they are reported and displayed in units of age in years or degree centrality, respectively.

**Figure 3.**
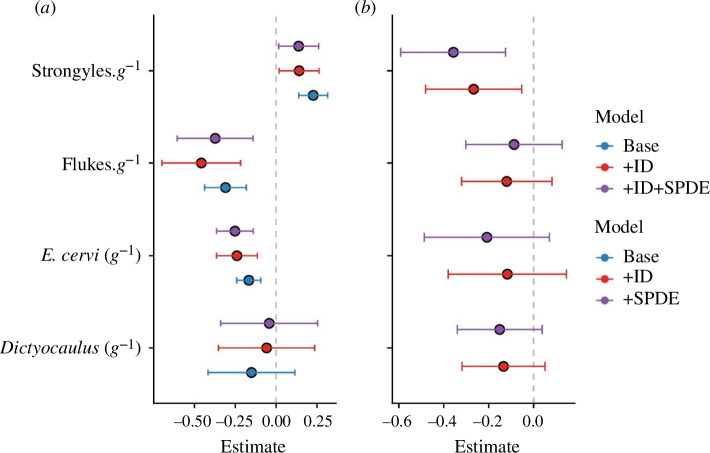
Model effect estimates for (*a*) the effect of age on parasite counts and (*b*) the effect of parasite counts on overwinter survival probability. Points represent the mean for each effect estimate; error bars denote 95% credibility intervals. All estimates are given on the link scale, in units of standard deviations. Different colours represent different model constructions: ‘+ID’ = includes a random effect of individual identity. ‘+SPDE’ = includes the ID effect, plus an additional spatially distributed stochastic partial differentiation equation random effect to account for spatial autocorrelation.

Spatial autocorrelation effects substantially improved the models for flukes and tissue worms (electronic supplementary material, table S1; ΔDIC < −3), but not for strongyles or lungworms (electronic supplementary material, table S1; ΔDIC > −2). These findings demonstrate that there was notable heterogeneity in parasite infection (electronic supplementary material, figure S2), but controlling for this effect did not impact our age estimates (figure 3*a*; electronic supplementary material, figure S1), demonstrating that changes in spatial location were unlikely to be responsible for our observed age effects. There were moderate density effects evident in the base models for *E. cervi* and *F. hepatica*, but these effects were removed when spatial autocorrelation was controlled for (electronic supplementary material, figure S1). The spatial distributions of these parasites largely agreed with earlier observations [46], with greater *F. hepatica* count in the south–middle of the study area and greater *E. cervi* count in a slow gradient moving towards the north, particularly the northeast (electronic supplementary material, figure S2).

In our behavioural models, we uncovered a moderate positive effect of degree centrality on strongyle infection (0.171, 0.052, 0.289, *p* = 0.005; see figure 2 for these represented on the data scale rather than in units of standard deviations). There were a number of effects that were initially significant in our non-spatial models but their effects were removed when spatial autocorrelation was accounted for (electronic supplementary material, figure S3), indicating that these behaviours were not possible to extricate from spatial heterogeneity in the parasite’s distribution. There was likewise a moderate effect of annual density on *F. hepatica* infection, which persisted when spatial autocorrelation was controlled for (−0.415, −0.796, −0.019, *p* = 0.04; electronic supplementary material, figure S3). In all cases, accounting for behaviours in the models had very little impact on the age estimates (electronic supplementary material, figure S3), demonstrating that age-related changes in parasitism were largely independent of behavioural effects.

We found that strongyle count was strongly associated with reduced overwinter survival probability (figure 3*b*; electronic supplementary material, figure S4; −0.98, −1.47, −0.55, *p* < 0.001), agreeing with previous findings [37]. This finding remained significant when spatial autocorrelation was controlled for (electronic supplementary material, figure S2). There were only weak negative non-significant trends with the other parasites (figure 3*b*; electronic supplementary material, figure S2; *p* > 0.05). Additionally, fitting random effects of individual identity substantially improved model fit (ΔDIC < −10; electronic supplementary material, table S1) but without notably affecting the age effect estimates (figure 3*a*). Taken together, these findings provide little evidence for a role of selective disappearance in *driving* our observations, except for potentially *obscuring* the age–strongyle trend. That is, our estimate for the age effect on the strongyle counts is a composite that likely includes a contrasting effect of selective disappearance, and is therefore likely an underestimate.

## 4. Discussion

We uncovered substantial and contrasting age-related changes in parasite count across different parasites in a long-lived wild mammal, which were not explained through considering behavioural or demographic factors. Ageing red deer experienced a small increase in strongyle nematode counts, which contrasted with stronger age-related decreases in liver fluke (*F. hepatica*) and tissue worm (*E. cervi*) counts. These findings add to a sparse body of longitudinal individual-based evidence for age-related changes in parasite count in wild animals [20,21,26,27,30,47]. Accounting for and quantifying spatial autocorrelation and fitting socio-spatial behavioural metrics in our models had no detectable effects on our age estimates, suggesting that these changes were unlikely to be driven by previously documented behavioural ageing patterns and resulting changes in exposure rate [10]. Similarly, there was no evidence that selective disappearance of certain individuals was driving our observed trends, given that survival costs were limited to strongyle infection and were insufficient to produce our observed trends. As such, these observations do not suggest that behavioural ageing drives age-related changes in parasite infection in this system, and instead imply that divergent age-related trends may arise for different parasites through changes in intrinsic (e.g. physiological or immunological) traits.

Our observation that greater social connectedness predicted greater strongyle count agrees with the conventional wisdom that infectious disease is a primary cost of sociality [48,49], but this trend was in the opposite direction to the direction we expected if social behaviour was playing a role in driving age–infection relationships. That is, if individuals’ ageing behaviour were driving the effect, because social connectedness decreases with age [10], we would expect strongyle count to likewise decrease with age. Instead, these findings are more suggestive of the reverse: ageing individuals may reduce their exposure to parasites as they decrease their social connectedness, which could ultimately minimize the effects of a waning immune system for strongyles. Indeed, this mechanism has been theorized several times [7,10], and recently received strong support via behavioural simulations [50]. If behavioural ageing is linked to reducing exposure owing to immunosenescence, because the strength of natural selection is expected to wane in later life [51,52], it is unlikely that this is an adaptive response specifically brought on by immunosenescence; instead, a relationship between behavioural ageing and infection could emerge through more general behavioural compensation for a weak immune response that evolved in earlier life and persists as the animal senesces. Such behavioural compensation is relatively common [53,54]: for example, Stephenson [55] demonstrated that guppies (*Poecilia reticulata*) show stronger conspecific avoidance when they are more susceptible to infection. Although it has yet to be shown that immunosenescence and social ageing are linked directly, our observations are consistent with a similar underlying process for strongyles. Conversely, although we noted a negative correlation between density and *F. hepatica* infection, and individuals tend to move to areas of lower density as they age [10], there was nevertheless a decrease in *F. hepatica* count with age. Therefore, behaviour was likewise countering age-related changes—but in the reverse pattern, by potentially driving *greater* exposure to *F. hepatica*—which were nevertheless counteracted by other phenotypic changes. Taken together, these findings indicate that behaviour likely plays a plastic or buffering role in mediating the relationship between phenotypes, age and infection as an individual ages.

It was also surprising that degree centrality—a social network metric—predicted strongyle count, rather than any spatial behaviour metrics. This effect was relatively strong, and corresponded to roughly a doubling in strongyle count across the range of degree centrality values (figure 2). This was perhaps unexpected as helminth parasites transmit indirectly, so we would expect that incorporating spatial measures (rather than more direct measures of social contact) may be more representative of indirect contact rates—and therefore of parasite counts [5]. For example, areas of higher density should be more intensely used and therefore support greater larval concentrations on the pasture. Further, the spatial autocorrelation effects in the models should account for age-related movements towards areas of variable transmission of certain parasites—for example, if lower *F. hepatica* counts were driven by movements away from wetter areas tend to support transmission via their water snail intermediate hosts [46]. Because social connections are parameterized according to spatiotemporal coincidence (i.e. they require individuals to be in the same location at the same time), the measures derived from this metric could be more indicative of between-individual helminth transmission, which could occur more on the timescale of days to months than years, even despite the fact that both social and spatial behaviours were ultimately summarized at the annual level. Regardless of the ultimate cause, these findings agree with the previous observation that social network position is both heavily intertwined with spatial behaviour in this system and a biologically important stand-alone measure [10,34]. This finding adds notably to the literature on spatial–social analysis in disease ecology, and accentuates the value of using both spatial and social metrics when quantifying the drivers of infection status [5].

Aside from behaviour, a variety of age-related changes could be responsible for divergent age trends among parasite taxa: on the immune side, increasing strongyle counts could be driven by decreased resistance brought about by immunosenescence, agreeing with previous observations in wild Soay sheep [21,56]. This observation disagrees with a previous finding that strongylid infection decreases with age in African elephants, for example [31]; given that that investigation occurred at the population level, it is possible that selective disappearance may have played a role in influencing this pattern in the elephants, accentuating the benefit of longitudinal individual-based studies for testing age–infection questions like these. Meanwhile, the decreasing *F. hepatica* and *E. cervi* counts could be indicative of acquired immunity over the lifespan, where older individuals become gradually more resistant owing to repeated exposure. This agrees with conventional wisdom in livestock that many ungulates can acquire an element of immunity to *F. hepatica* infection [57], but disagrees with observations of increased *F. hepatica* prevalence in older age categories taken from wild studies [58].

It is unclear how and why age-related trends would diverge for strongyles compared to *F. hepatica* and *E. cervi*, and why acquired immunity might play a greater role for the latter two rather than the former. Confirming a role for immunity would require (i) measuring a suite of immune traits to examine how they change with age, and (ii) examining whether they correlate with parasites and could therefore represent immune resistance (i.e. the ability to reduce parasite load) [59]. Given that the strongyle counts were measured at the order level, and generally comprise a mixture of different species, one possibility is that even within this parasite count there is age-related change in the community, with certain species dominating in early years that are then replaced by higher-intensity infections with other species. Related to this, coinfecting parasites could interact with each other, either facilitating or preventing each other establishing an infection in ways that contribute to the age-related changes we observe [60]. For example, if strongyles and tissue worms compete indirectly by invoking the same immune responses, age-related increases in strongyle intensity could result in a concurrent decrease in tissue worm count. Confirming community-level changes like these would require more precise taxonomic identification of the constituent nematodes, e.g. through DNA-based approaches [61,62]. A similar trend is less likely for the fluke and tissue worm counts, as these are more likely to be counts of single homogenous species. Finally, another option—given that strongyles reside in the gut, unlike the other two—is that these changes are mediated by gut-specific changes with age, for example in microbiota composition. Ultimately, the fact that these reputedly similar macroparasites showed highly divergent trends with age is interesting, and invites further investigation.

Overall, our results confirm that age-related changes in infection can vary substantially within the same system, and likely depend on a complex combination of immune, behavioural, and demographic processes. Although we did not test specific immunological drivers of the trends we observed, this study suggests that changes in exposure and demography through the lifespan could play a complex role in age–infection interrelationships, and that changes in intrinsic (i.e. physiological) traits might be relatively more important. Given the highly divergent age trajectories observed, this study confirms that ageing individuals may not necessarily experience a greater overall parasite burden, but a different parasite community, which may exert complex pressures on the age structure of the population. Understanding how and why parasite community structure changes with host age—and the relative role of susceptibility and exposure in determining it—is likely to provide new insight into parasite transmission and the ageing process in natural systems.

## Data Availability

Code and data are archived on Zenodo [63]. Supplementary material is available online [64].
